# Unveiling Distinctive Eye Tracking Markers to Distinguish Toddlers with High-Risk Autism as Indicated by ADOS Within an Elevated-Likelihood Toddler Sample

**DOI:** 10.3390/children13010055

**Published:** 2025-12-30

**Authors:** Orsolya Pachner, Péter Soltész, Ferenc Gombos, Patrícia Gerván

**Affiliations:** 1 Institute of Education and Psychology at Szombathely, ELTE Eötvös Loránd University, 1075 Budapest, Hungary; 2 Roska Tamás Doctoral School of Sciences and Technology, Pázmány Péter Catholic University, 1088 Budapest, Hungary; soltesz.peter@btk.ppke.hu; 3 Institute of Psychology, Pázmány Péter Catholic University, 1088 Budapest, Hungary; 4 Laboratory for Psychological Research, Pázmány Péter Catholic University, 1088 Budapest, Hungary; gombos.ferenc@btk.ppke.hu; 5 Institute of Psychology, ELTE Eötvös Loránd University, 1075 Budapest, Hungary; gervan.patricia@ppk.elte.hu

**Keywords:** autism spectrum disorder, toddler, eye tracking, social attention

## Abstract

Background: Eye tracking technology can be utilized to identify early markers of autism. Several neurodivergent features of social attention have been revealed by eye tracking studies in ASD toddlers. Our aim was to develop stimulus material that elicits highly distinctive gaze patterns in toddlers at low and high risk for autism as indicated by ADOS (i.e., scoring below and above the ADOS cut-off point). Additionally, we sought to identify the variables most effective in differentiating between these groups. Methods: In our research, we analyzed the data of 74 toddlers between 12 and 30 months. Children were divided into two groups based on their ADOS scores: the lower ADOS (lrADOS) group included those scoring below the ADOS cut-off point (*n* = 42; mean age = 22.5 ± 5.0 months), while the higher ADOS (hrADOS) group comprised children scoring above the cut-off (*n* = 32; mean age = 23.3 ± 4.8 months). We recorded eye gaze data during the presentation of dynamic social attention stimuli with a self-developed eye tracking device. We used two types of social attention stimuli: joint attention (ostensive) and preferential looking paradigm. We analyzed the area of interest based on the gaze–time ratio. To ensure sufficient robustness, we selected gaze retention interval (GRI) variables characterized by broader spatial and temporal parameters compared to traditional fixation-based measures. Results: As anticipated, we observed significant differences between the two groups across most variables. Typically, within the preferential paradigm, the distinct indicators of the social domain were higher on average in the lrADOS group compared to the hrADOS group, while the non-social domain exhibited the opposite trend. The results of correlations with ADOS scores indicated that the social ratio in the preferential paradigm exhibited the strongest negative correlation. Notably, there were higher effect sizes within the hrADOS group in comparison to the lrADOS group when correlation with ADOS scores was tested separately for each group. Conclusions: We developed stimulus materials and eye tracking variables that, thanks to their robustness, appear promising even when dealing with noisy eye tracking data typical of young children. In the preferential paradigm, beyond AOI ratio measures, GRI variables show promise in distinguishing between toddler groups with higher vs. lower ADOS scores. Furthermore, they may be related to severity based on their marked and significant correlations with ADOS scores. Especially when used in combination, these variables appear well-suited to capturing characteristics indicative of an elevated likelihood of autism.

## 1. Introduction

Eye tracking methods have gained popularity in autism spectrum disorder (ASD) research over recent decades [1,2,3]. Previous studies have demonstrated that eye tracking technology can identify various characteristics of ASD, ranging from behavioral patterns to neurocognitive functions [1]. It is also a suitable method for use with infants and toddlers. However, conducting eye tracking research in early childhood presents several challenges, including limited attention spans, the need for movement, and a restricted understanding of instructions [4]. Despite these difficulties, numerous studies have found consistent differences in eye tracking data between toddlers with and without ASD [5,6,7,8,9,10,11,12,13,14,15,16,17], raising the possibility of early detection.

One of the most extensively researched areas in ASD is social attention [9]. Individuals with autism are known to exhibit reduced attention to social stimuli as compared to non-social stimuli. This social attention deficit is further characterized by difficulties in processing eye gaze information and other ostensive cues, particularly in joint attention situations. Although these two facets are related, research often examines them using distinct but overlapping paradigms [10].

Reduced attention to social stimuli has been studied using the preferential looking paradigm, in which two videos (or images) are presented side-by-side—one depicting socially relevant content and the other a non-social stimulus (e.g., objects or geometric shapes) [12,15]. One of the most widely studied paradigms of this kind is the GeoPref test. Pierce and colleagues [13,14,17] demonstrated that increased visual attention to geometric images over social images may serve as a potential stratification marker for a specific subgroup of toddlers with autism. In their most recent study [17], they reported highly replicable and reliable results. This candidate stratification marker was particularly effective in identifying toddlers with ASD who exhibit more severe symptoms, as well as lower language and cognitive abilities. In the present study, the objective was partly similar to identify potential markers for autism in toddlers. However, the present study’s primary focus was on the elevated-likelihood sample as opposed to toddlers with an autism diagnosis who exhibited severe symptoms. The primary challenge in the early detection of autism, apart from the identification of children with mild to moderate symptoms, is the differential diagnosis. The detection of eye-tracking signs associated with autism in a sample of elevated likelihood would represent a significant step towards resolving this challenge.

In contrast, the joint attention paradigm has been applied less consistently across studies. Typically, these paradigms involve video stimuli in which an adult attempts to engage the toddler’s attention and then directs their gaze toward an object. As in the preferential looking paradigm, there are also social stimuli (the adult) and non-social stimuli (the objects), but in these videos, the interaction is an additional element. The person in the video tries to interact with the toddler and draw their attention to an object. Due to the testability of joint attention, there are two objects, one of which is at the center of shared attention. Research in this area explores various aspects such as responding to [5,6,7], initiating [6,11], or the effect of labeling stimuli [8,16]. Some studies also focus on preferences for the eye or mouth regions of the face. Findings regarding eye region fixation show no consistent differences between toddlers with ASD and those with typical development (TD) [8,16], and results for the mouth region are similarly mixed [16,18]. In terms of response behaviors, Chawarska, Macari, and Shic [7] found that attention in toddlers with ASD decreased when an adult initiated joint attention in video scenes, particularly when the adult used more ostensive cues (e.g., child-directed speech and eye contact). Their results suggest that reduced attention to faces in ASD is context-dependent. Other studies [5,6] found no clear group differences in response tasks such as gaze following. In measures of initiation, differences in eye tracking data have been observed in toddlers with ASD, especially in transition patterns between looking at objects and faces [6,11].

It should be noted that other non-social cognitive mechanisms have also been examined using eye trackers. For instance, attentional disengagement is a pivotal cognitive process which exhibits considerable variance in individuals diagnosed with ASD and other neurodevelopmental conditions, extending from infancy through to adulthood. This attention control is also a valuable mechanism that can underlie the results of an eye tracking study using a social attentional paradigm [1,19]. It is hypothesized that the implementation of two distinct social attentional paradigms—preferential looking and joint attention—will facilitate the capture of multiple attentional functions in the background.

In addition to attention control, oculomotor disturbances may also be a contributing factor to the results obtained with eye tracking methods. According to a meta-analysis [20], there are several oculomotor disturbances in autism: saccade dysmetria, deficits in pursuit eye movement, more frequent and larger intrusive saccades, and thus disturbances in the maintenance of stable fixation. However, the majority of the analyzed articles focus on adults, with only one article focusing on toddlers. Two recent studies have focused specifically on oculomotor differences in children aged 1–11 [21,22]. In the study of Avni et al. [21], the majority of children diagnosed with ASD demonstrate similar oculomotor function to the control group. They suggest that social gaze abnormalities do not stem from fundamental oculomotor deficiencies. In contrast, Ziv et al. [22] found that oculomotor randomness was greater in the ASD group than in the TD group. In summary, the study observed a greater prevalence of noisy and random eye movements in children diagnosed with ASD compared to the TD group. A further valuable insight derived from their study was the observation that the quality of the recordings from the ASD group was found to be lower in comparison to those from the TD group. In this context, it should be noted that Avni et al. [21] excluded half of their initial sample from their analyses due to missing data. This is a common practice in the field of eye tracking studies, which can result in a general bias.

In order to respond to this limitation, an analytical method was developed with the objective of including as many participants as possible in the analysis. The relevant details are presented in the methodology section. Here, we would like to highlight the creation of variables based on gaze retention intervals, which reflect previous oculomotor differences in ASD and work with a broader time and space boundary than fixations. It is hypothesized that this will reduce data gaps resulting from minor oculomotor disturbances, while allowing social attention skills to be studied in a broader toddler group.

The aim of our study was to examine two types of social attention paradigms by analyzing specific eye tracking variables in toddlers below and above ADOS cut-off points, since studies found an elevated likelihood for ASD. We conducted our research using a custom-designed eye tracking device tailored for studying young children. Our primary research question was whether eye movement patterns in this combined prevalent social attention paradigm set could effectively differentiate toddlers with ADOS scores above the cut-off point from those in an elevated-likelihood group.

## 2. Materials and Methods

### 2.1. Participants

Participants were recruited through social media advertisements and various early childhood institutions, such as primary care facilities and diagnostic centers. During recruitment, we specifically highlighted whether concerns regarding autism had been raised by either parents or professionals working with the child. As a result, most parents had some suspicion or curiosity about autism, which means the sample was drawn from an elevated-likelihood population rather than the general population.

Eighty parents of toddlers applied for our research call. From the final sample, we excluded 6 toddlers (7.5%). Subjects who had a visual acuity problem of 4+ dioptres (*n* = 1), who could not be tested with ADOS (*n* = 3), or who failed the eye movement tracking task (*n* = 2) were excluded from the experiment. It is important to emphasize that we excluded only those who could not be seated in front of the screen at all (not even on their mother’s lap). We defined the criterion for exclusion due to data loss as 50% of the total stimulus duration, but there were no such cases in the study sample.

### 2.2. Procedure

Parents registering for research filled out a questionnaire about their child’s anamnestic data (e.g., developmental problems) online. The child’s participation began with the eye tracking procedure (max. 5 min), followed by ADOS-2 and ophthalmological screening (the two together max. 45–60 min). The procedures were conducted by two specialists, with the ADOS assessment being conducted by the specialist who was blind to the results of the eye tracking procedure. The parent completed the M-CHAT questionnaire during these examinations. Families received a 5000 HUF (approx. 14.2 USD) voucher upon their participation.

### 2.3. Clinical Measures

ADOS-2 Toddler Module

The Autism Diagnostic Observation Schedule–2 (ADOS-2) is a semi-structured, standardized assessment tool used for diagnosing autism spectrum disorder (ASD). In this study, we utilized the Toddler Module, which is designed for children under 30 months of age and includes tasks similar to those in Module 1 of the original ADOS [23].

We applied two different diagnostic algorithms and cut-off scores, depending on the verbal ability of the child. The non-verbal algorithm was used when toddlers’ general language level during the assessment was limited to fewer than five words or who had no spontaneous words at all. The cut-off scores were determined based on the work of Hong et al. [24], which compared ADOS-2 scores and subsequent and independent ASD diagnosis. The algorithms, which calculate the scores of particular items from the measure, provide a classification indicative of autism, ASD or nonspectrum conditions. It is noted that two distinct algorithms have been identified, given that items pertaining to speech are considered irrelevant for non-verbal children. For nonverbal children (Algorithm 1), a cut-off score of 10 yields 99.15% specificity, with 92.13% positive predictive value and 95.35% negative predictive value. For verbal children (Algorithm 2), the cut-off score was 8, with 97.10% specificity, 88.81% positive predictive value, and 94.44% negative predictive value. Based on these thresholds, participants were categorized into lower ADOS (lrADOS) and higher ADOS (hrADOS) groups.

All assessments were conducted by specialists certified in the administration of the ADOS-2. To evaluate interrater reliability, a subsample of 10 toddlers (mean age = 23.5 months; SD = 4; including 3 lrADOS and 7 hrADOS) were independently rated by both our specialists and an experienced clinical psychologist. Interrater agreement on the algorithm items ranged from 86% to 100%, with a mean agreement of 93.6%. There was only one discrepancy in diagnostic classification between the two raters (κ = 0.737). Based on this substantial agreement, all subsequent ADOS assessments were performed by our own certified specialists.

However, as ADOS is not sufficient on its own for clinical diagnosis in Hungary, if the ADOS score indicated the likelihood of ASD, the parents were referred to a child psychiatrist for further assessment.

Ophthalmological Screening

Ophthalmological screening was conducted to identify common visual issues that may accompany neurodivergent development. The screening procedures were administered by our examiners, who were trained by a specialist in pediatric ophthalmology and strabismus. The tests included guided eye movement, cover and uncover test, and the Lang II stereotest. In five cases, symptoms indicating mild strabismus were observed. In one case, the child exhibited symptoms consistent with mild myopia. However, it should be noted that 29 children were uncooperative during the ophthalmological examinations at the end of the procedure.

M-CHAT

The Modified Checklist for Autism in Toddlers (M-CHAT) is a standardized parent-report screening tool designed to identify children aged 18–24 months who may be at likelihood for autism [25]. In this study, the M-CHAT was administered to all participants aged 12–30 months, as it is a non-invasive measure that provides relevant developmental insights even outside the standard age range. We did not analyze these data in our study; we only used them to confirm the ADOS diagnostic indication, in order to refer suspected ASD cases for further clinical evaluation.

### 2.4. Eye Tracking Methods

Eye Tracker

We used a custom-built eye tracking device developed by ADDAS Ltd. (Budapest, Hungary) research team. The system consists of a uEye UI-3860LE-M monochrome camera (IDS Imaging Development Systems GmbH, Obersulm, Germany) (featuring a SONY IMX-290 sensor (Sony Semiconductor Solutions Group, Atsugi, Japan)) equipped with 16 mm optics (Tamron Co. Ltd., Saitama, Japan) and four 850 nm infrared LED emitters (Elektro Meier, Gliching, Germany). These emitters are arranged in a rectangular formation, approximately 5 cm inward from each corner of the virtual stimulus presentation area. The infrared illumination and visual stimuli are optically merged using a selective-wavelength semi-transparent mirror.

This self-developed eye tracker was designed specifically for infant and toddler research. It tolerates head movement and does not require calibration, a key advantage when working with children under the age of 3 who may find it difficult to follow instructions. Omitting the calibration step significantly reduces participant exclusion due to non-compliance.

During data collection, toddlers were seated on their caregiver’s lap at a distance of approximately 50 cm from the device housing, 100 cm from the front lens, and 110 cm from the LED emitters. The system recorded at 50 frames per second with a resolution of 1936 × 1096 pixels in a single channel. Independent laboratory testing confirmed that the infrared LEDs emit safe levels of radiation for both the cornea and retina, even at the minimum subject distance of 50 cm and across the full range of expected movement.

Our eye tracker is optimized for the natural head movement typical in infants. Head movement is tolerated as long as at least one eye remains within the camera’s field of view, which depends on the subject’s distance from the camera. The optics are calibrated to allow head movement up to 30 cm forward and 10 cm backward from the original position while still maintaining reliable gaze capture. Backward movement is naturally constrained by the child being seated in the caregiver’s lap.

We evaluated the device’s accuracy and precision using a sample of 11 adults with normal vision. On a central fixation target, average accuracy was 0.74° in the X direction and 1.25° in the Y direction, while average precision was 0.37° (X) and 0.53° (Y). It is acknowledged that the accuracy may differ in toddlers; thus, larger AOIs were selected partly to accommodate this.

The device outputs raw gaze position data, computed based on binocular eye positions. Missing gaze rate was calculated on data-points where any meaningful stimulus occurred concurrently, on a subject basis. The median missing gaze rate was 9.5%. This percentage also covers blink related missing gaze. In cases where only one eye was visible or detectable, gaze points were estimated using a model-based algorithm that predicts the binocular gaze from the visible eye, based on a previously trained relationship. Median prevalence of gaze calculation from single eye data was 29.2%.

Stimuli and presentation

Conducting eye tracking research with toddlers presents several challenges, including limited attention spans, high movement needs, and a limited understanding of instructions. To address these issues, two iterative pilot phases preceded the current study (*n*_1_ = 34; *n*_2_ = 35). These phases were used to select the most suitable stimulus types and items from a broader set of materials. Additionally, we tested the feasibility of our eye tracking protocol on typically developing toddlers.

**Figure 1:**
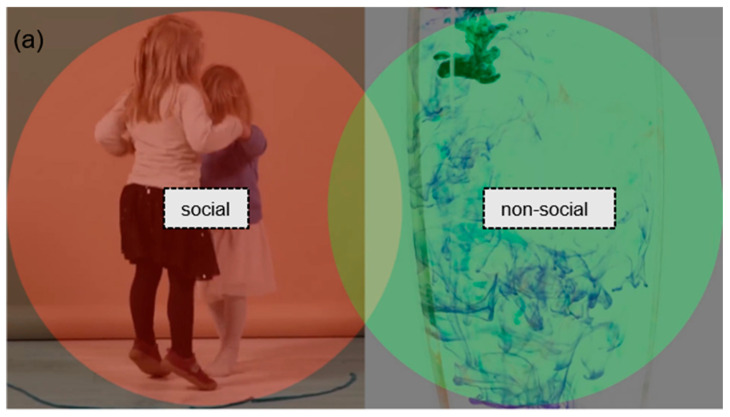

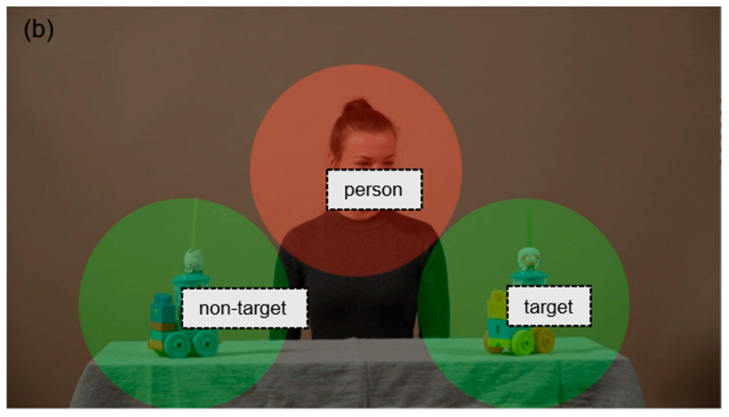
Examples of (**a**) preferential and (**b**) ostensive stimuli with AOI markings.

Based on the pilot results, we selected two primary types of stimuli for the main study: joint attention (ostensive) scenes and preferential looking stimuli that contrasted social and non-social content (see Figure 1 for examples).

In the ostensive video stimuli, an actress first engaged in ostensive communication by looking into the camera, smiling, and addressing the child (e.g., saying “hello”). She then directed the child’s attention to one of two similar objects (toys) by shifting her gaze and head. In total, there were seven of this type, with durations ranging from 9 to 13 s.

In the preferential looking stimuli, two dynamic videos were presented side-by-side. One side always featured a social video, which could include an adult using ostensive communication, a child moving, or a group of children interacting (e.g., dancing or playing together). The other side showed a non-social video, consisting of moving objects or abstract, nonfigurative scenes. All videos were real-life recordings. The side on which the social scene appeared was counterbalanced across trials. There were 16 videos of this type, with durations ranging from 8 to 13 s.

The areas of interest were defined using the Limited Radius Voronoi Tesselation method. In order to establish preferential stimuli, social (red) and non-social (green) areas have been defined. In the case of ostensive stimuli, the person is represented by the social relevant area (red); the areas of objects—target and non-target—are green.

The videos were presented in a quasi-random order. During the presentation, the videos began with animation (zoom-out) and finished with animation and a sound signal (zoom-in and short beep). They were used to keep the children’s interest and direct the children’s attention to the screen. We excluded these parts from the analysis—with the exception of the gaze stuck variable which focuses specifically on the analysis of the zoom-out sections.

Eye tracking variables
AOI variables

The areas of interest (AOI) were developed using the Limited Radius Voronoi Tesselation (LRVT) method [26]. In preferential stimuli, the social and non-social parts were defined in space and time, and in ostensive stimuli, there was a person, a target, and a non-target AOI (see examples in Figure 1). The videos were sectioned into temporal parts that represented similar situations across multiple similar stimuli. In the preferential paradigm, we used only the core part of the stimuli for to define the AOI without the animations at the beginning and the end. In the ostensive stimuli, we used only the postturn-towards-target parts of videos. Our AOI variables relied on the gaze–time ratio. We used five different AOI variables. In preferential situations, the ratio of social (proportion of time an individual spends looking the social AOI, in %) and ratio of non-social (proportion of time an individual spends looking the non-social AOI, in %) and, in ostensive situations, the ratio of person (proportion of time an individual spends looking the person AOI, in %), ratio of target (proportion of time an individual spends looking the target AOI, in %), and ratio of non-target (proportion of time an individual spends looking the non-target AOI, in %) variables were analyzed.

Gaze retention interval (GRI) variables

To create sufficiently robust variables, we opted for gaze retention interval (GRI) variables with a broader spatial and temporal range as compared to the classical fixation variable. In research investigating fixations in young children, the subject exclusion rate was 11–26%, which was frequently due to a significant (40–50%) lack of data [17,18,27]. In a meta-analysis, Von Holzen and Bergmann [28] reported dropout rates ranging from 0 to 69% in mispronunciation detection studies with one-to-four-year-olds using the preferential looking paradigm. For detecting the gaze retention interval (GRI), a common algorithm was used, the identification by dispersion threshold (IDT) algorithm [29,30,31,32]. With the conventional parameter settings, the algorithm detects fixations. However, with modified (more tolerant) parameters, it is able to detect the more dispersed, longer duration GRI. The IDT algorithm uses the x- and y-data of the eye positions and two fixed thresholds: the maximum fixation dispersion and the minimum fixation duration. To be a GRI, eye movement data samples have to be within a spatial area not exceeding the dispersion threshold (300 pixels) for at least the duration threshold (300 ms). Since participants were able to move towards or away from the monitor slightly, the fixation areal threshold was set so that at minimum it represents 8° at 600 mm distance and 6° at 800 mm distance, which corresponds to approximately 300 pixels on the monitor in both directions. The samples fulfilling these criteria were marked as GRIs. Based on GRI detections, we calculated density (number of GRI in a minute), duration (average length of GRI in msec), gaze time (duration of all GRI in msec), gaze stuck (duration of GRI in msec after the end of an AOI), GRI in AOI ratio (number of GRI in an AOI/number of all GRI during the AOI time), and latency (time in msec from the start of an AOI to the first GRI in the AOI) parameters. In addition to the variables commonly implemented in studies employing fixations (e.g., density, duration, and latency), we also applied less common variables (e.g., gaze stuck). The gaze stuck variable was employed to specifically capture the phenomenon of attention disengagement, which was examined by quantifying the duration of gaze on the previous GRI area after the end of the AOI. Latency also attempts to capture this aspect at the beginning of AOIs. The GRI in the AOI ratio differs from the simple AOI ratio in that it provides more detailed information about eye movements within the AOI area.

For each subject, the values of the eye movement and gaze retention interval (GRI) variables were calculated in the preferential and ostensive situations in each AOI for each stimulus. The values of variables from different stimuli but belonging to the same AOI were averaged. This resulted in robust variables where the effects of occasional missing data due to not watching the stimuli were mitigated.

### 2.5. Statistical Method

To determine the optimal sample size, we performed power calculations for independent sample tests. A sample size of *n* ≥ 34 was found to be required for a power of at least 0.8 to detect a 0.7 effect at *p* < 0.05. Therefore, we decided that our sample size would be near *n* = 34 in the lrADOS and hrADOS groups.

The lrADOS and hrADOS groups were compared used either Student tests, Welch tests, or Mann–Whitney tests. Normality was tested using a Shapiro–Wilk test for both groups, but the assumption of normality was held up if absolute skewness was below 1 and absolute kurtosis was below 2. If normality held up but equality of variances was violated (tested by Levene’s test), a Welch test was used. If either group violated the assumption of normality, a Mann–Whitney Test was used. Hedge’s g was used to calculate the effect size of the differences between the groups

For correlations, the assumption of normality was checked by a Shapiro–Wilk test in a pairwise manner; furthermore the assumption of normality was held up if absolute skewness was below 1 and absolute kurtosis was below 2. Linearity and homoscedasticity were visually checked. If any of these assumptions failed, a Spearman correlation was conducted (with a further visual test on monotonicity), otherwise a Pearson correlation was calculated.

The difference (expressed in Fisher’s z score) between the correlations in the lrADOS and hrADOS groups and the significance of the difference was also calculated. The Holm–Bonferroni method of false detection rate correction was performed to correct for multiple comparisons. Statistical tests were carried out in G*Power 3.1.9.7 software (Franz Faul, Univedrsitat Kiel, Germany, 1992–2020) for power analysis. TIBCO STATISTICA 14.0.1.25 software (TIBCO Software Inc., Palo Alto, CA, USA 1984–2020) was used for independent sample tests and correlation calculations. Microsoft Excel Professional 2021 was used for Hedge’s g effect size and Holm–Bonferroni calculations. An online tool (VassarStats: Website for Statistical Computation, Richard Lowry 2001–2023; http://vassarstats.net/rdiff.html (accessed on 10 September 2023.)) was used to compute the significance of the difference between two correlation coefficients using the Fisher r-to-z transformation.

## 3. Results

### 3.1. Participant Characteristics

The final sample of 74 toddlers (aged from 12 to 30 months with M = 22.8 and SD = 4.9) met our inclusion criteria for the study. We classified toddlers into two subgroups based on their ADOS scores. A total of 43% of our sample was above the cut-off point. In the lrADOS subgroup, we included those with ADOS scores below the cut-off points (*n* = 42). For a detailed description of the study population, see Table 1. It is important to note that the sample included twice as many male subjects as female subjects. Different algorithms (Algorithm 1 or Algorithm 2) were used to determine the ADOS score depending on the age and speaking ability of the subjects. The mean age was 20.8 months for Algorithm 1 (*n* = 42; hrADOS ratio = 57.1%) and 25.5 months for Algorithm 2 (hrADOS ratio = 25.0%).

### 3.2. Statistical Analysis

#### 3.2.1. Differences Between lrADOS and hrADOS Groups

Differences in eye movement variables between the lrADOS and hrADOS groups were tested using independent samples tests, and Hedge’s g effect sizes were also calculated.

AOI variables

**Figure 2:**
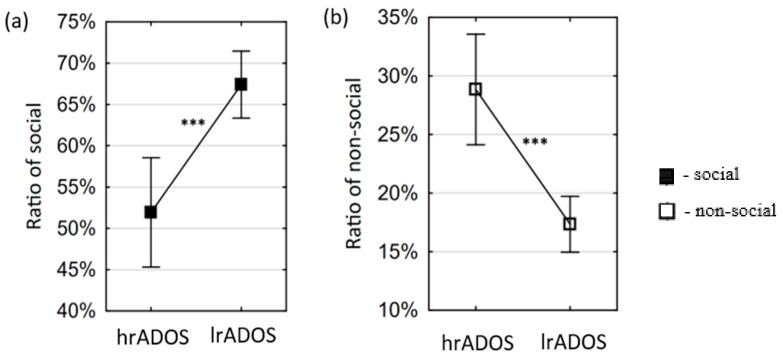
Differences between lrADOS and hrADOS groups in eye movement variables in preferential situations. Small squares represent the mean of the eye movement variables, and error bars depict 95% confidence intervals of the mean. Asterisks indicate significant differences (***: *p* < 0.001). The mean of the hrADOS subjects is significantly higher than the mean of the lrADOS subjects in the variables of social ratio (**a**), while it is significantly lower in the variables of non-social ratio (**b**).

For the preferential situations, the mean of the hrADOS subjects is significantly lower (t(71) = −4.2369; *p* = 0.0001) than the mean of the lrADOS subjects in the variable of social ratio (Figure 2a). In align with this, the opposite pattern (t(71) = 4.7094; *p* = 0.0000) is visible in case of the non-social variable (Figure 2b). For the ostensive situations, the mean of the hrADOS subjects was lower than the mean of the lrADOS subjects in the variable of ratio of person (t(51.49) = −2.0764; *p* = 0.0429). Hedge’s g effect sizes were large for both significant differences in the preferential situations and moderate for the significant difference in the ostensive situations (Table 2). For the preferential situations, all differences remained significant after correction for multiple comparisons; however, for the ostensive situations, the differences did not survive the stringent correction for multiple comparisons (see OSF Data Repository, OSFDR).

GRI variables

**Figure 3:**
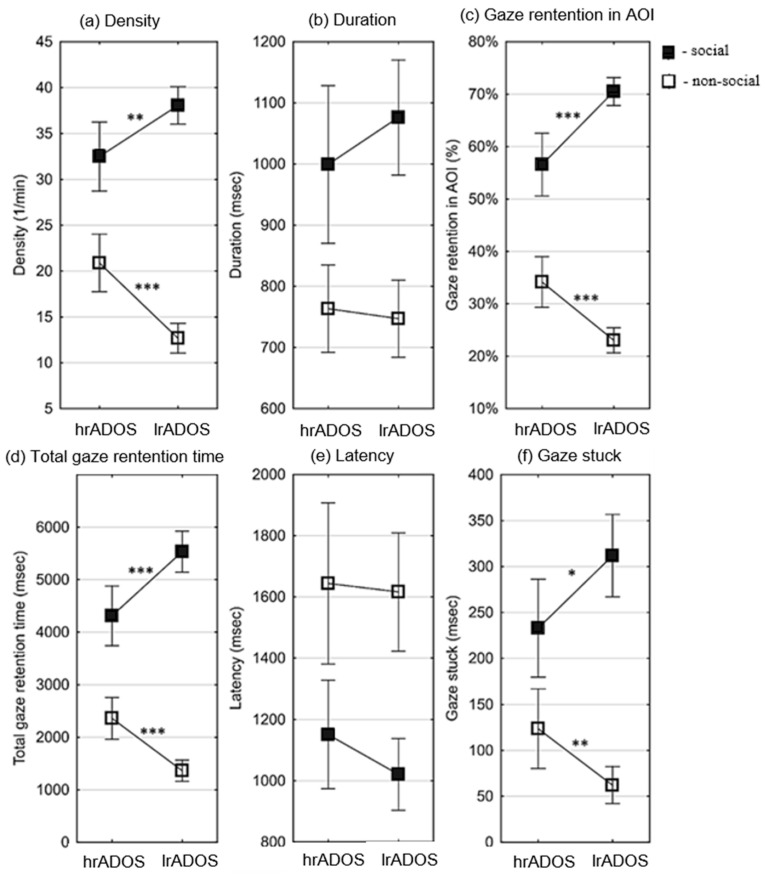
Differences between lrADOS and hrADOS subjects in gaze retention interval (GRI) variables in preferential situations. Small squares represent the mean of the eye movement variables, and error bars depict 95% confidence intervals of the mean. Asterisks indicate significant differences (*: *p* < 0.05; **: *p* < 0.01; ***: *p* < 0.001). The results for the social ranges are depicted by filled, and those for the non-social ranges are depicted by un-filled markers. For the variables of density (number of GRI in a minute) (**a**), gaze retention in AOI ratio (number of GRI in an AOI/number of all GRI during the AOI time) (**c**), total gaze retention time (duration of all GRI in msec) (**d**), and gaze stuck (duration of GRI in msec after the end of an AOI) (**f**), the mean of the hrADOS subjects was significantly lower in the social ranges and significantly higher in the non-social ranges than the mean of the lrADOS subjects. There was no significant difference in duration (average length of GRI in msec) (**b**) and latency (time in msec from the start of an AOI to the first GRI in the AOI) (**e**).

Differences in GRI variables in preferential situations between the hrADOS and lrADOS groups were tested using independent samples tests, and Hedge’s g effect sizes were also calculated (see Table 3). For the variables of density, gaze retention in AOI, total gaze retention time, and gaze stuck, the mean of the hrADOS subjects was significantly lower in the social ranges and significantly higher in the non-social ranges than the mean of the lrADOS subjects (see Figure 3). Hedge’s g effect sizes were large/very large for all significant differences except for gaze stuck in social situations (moderate effect). All differences remained significant after correction for multiple comparisons, except for gaze stuck in social situations (see OSFDR).

#### 3.2.2. Correlation with ADOS Scores

AOI variables

Correlations between ADOS scores and eye movement variables were computed in the group of all subjects. In preferential situations, a positive correlation was found between the ADOS score and the non-social ratio (r = 0.59; *p* < 0.0001), and a negative correlation was found between the ADOS score and the social ratio (r = −0.63; *p* < 0.0001). In ostensive situations, a negative correlation was found between the ADOS score and the variable of person ratio (r = −0.41; *p* = 0.0004). All significant correlations remained significant after correction for multiple comparisons (see OSFDR).

**Figure 4:**
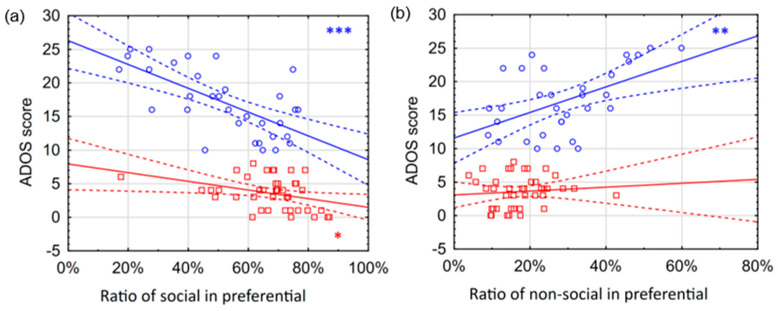
Correlations between ADOS scores and AOI variables in preferential looking situations. The figure separately shows the correlations between the ADOS score and different eye movement variables for hrADOS (blue circles) and lrADOS (red squares) subjects. Bold lines denote a linear correlation with the associated confidence interval (area between dashed lines). Blue (hrADOS) and red (lrADOS) asterisks indicate significant correlations (*: *p* < 0.05; **: *p* < 0.01; ***: *p* < 0.001). The correlations are most evident for hrADOS subjects. Social ratio variable shows a strong negative correlation (**a**), while non-social ratio positively correlated with the ADOS score (**b**).

In addition, correlations between ADOS scores and eye movement variables were examined separately in the group of hrADOS and lrADOS subjects. The difference (expressed in Fisher’s z score) between the correlations in the lrADOS and hrADOS groups and the significance of the difference were also analyzed (see last two columns in Table 4). In the lrADOS group in preferential situations, a weak negative correlation was found between the ADOS score and the variable of social ratio (r = −0.35; *p* < 0.05) (Figure 4a). No significant correlation was found in the ostensive situations. In contrast, in the hrADOS group with preferential situations, a strong positive correlation was found between the ADOS score and the non-social ratio (r = 0.51; *p* = 0.003) (Figure 4b). Alongside with this, a strong negative correlation was found between the ADOS score and the variable of social ratio (r = −0.66; *p* < 0.0001) (Figure 4a). In ostensive situations, a negative correlation was found between the ADOS score and the variable of person ratio (r = −0.45; *p* = 0.0092). The differences between the correlations of hrADOS and lrADOS subjects were not significant. All significant correlations did survive the stringent correction for multiple comparisons (see OSFDR). A partial correlation analysis was performed to adjust for the effects of age, and it was found that all subsequent correlations retained approximately equivalent effect sizes (see OSFDR).

GRI variables

Correlations between ADOS scores and GRI variables were examined in the group of all subjects in preferential situations. A significant correlation was found between the ADOS score and the variables of density, gaze retention in AOI, total gaze retention time and gaze stuck (|r| between 0.3 and 0.68). The correlations were positive in non-social areas and negative in social areas. In contrast, latency correlated positively in social areas (Table 5).

**Figure 5:**
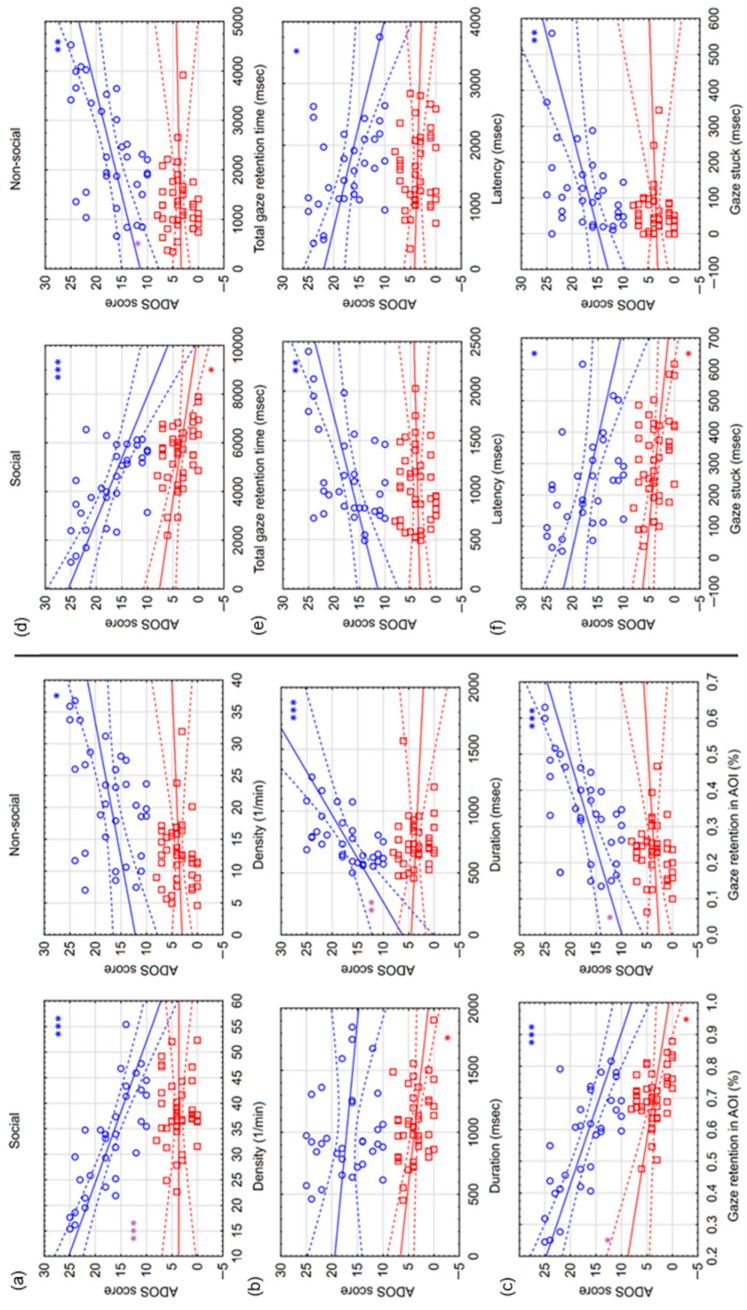
Correlations between ADOS scores and gaze retention interval (GRI) variables in preferential situations. The figure shows the correlations between the ADOS score and different GRI variables separately for hrADOS (blue circles) and lrADOS (red squares) subjects. Bold lines denote a linear correlation with the associated confidence interval (area between dashed lines). Blue (hrADOS) and red (lrADOS) asterisks indicate significant correlations (*: *p* < 0.05; **: *p* < 0.01; ***: *p* < 0.001). Purple asterisks denote significant differences between the correlations of hrADOS and lrADOS subjects. The subplots show correlations with density (number of GRI in a minute) (**a**), duration (average length of GRI in msec) (**b**), gaze retention in AOI ratio (number of GRI in an AOI/number of all GRI during the AOI time) (**c**), total gaze retention time (duration of all GRI in msec) (**d**), latency (time in msec from the start of an AOI to the first GRI in the AOI) (**e**) and gaze stuck (duration of GRI in msec after the end of an AOI) (**f**). For each variable, correlations for social and non-social areas are listed side by side.

In addition, correlations between ADOS scores and GRI variables were examined separately in the group of hrADOS and lrADOS subjects. The difference (expressed in Fisher’s z score) between the correlations in the lrADOS and hrADOS groups and the significance of the difference were also analyzed (Table 5). In the lrADOS group in social areas, a weak negative correlation was found between the ADOS score and the variables of duration, gaze retention in AOI, total gaze retention time and gaze stuck (r between 0.35 and 0.38). In the hrADOS group, a strong significant correlation was found between the ADOS score and the variables of density, gaze retention in AOI, total gaze retention time and gaze stuck (r between 0.41 and 0.77). The correlations were positive in non-social areas and negative in social areas. In contrast, latency correlated positively in social areas and negatively in non-social areas. Duration correlated only in non-social areas, and the correlation was positive (Figure 5). The difference between the correlations of hrADOS and lrADOS subjects was significant in non-social areas for the variables of duration, gaze retention in AOI and total gaze retention time and in social areas for the variables of density and gaze retention in AOI. All significant correlations in the group of hrADOS subjects remained significant after correction for multiple comparisons; however, significant correlations in the group of lrADOS subjects did not survive the stringent correction for multiple comparisons (see OSFDR). A partial correlation analysis was performed to adjust for the effects of age, and it was found that the majority of the correlations retained a comparable effect size. The correlation between the ADOS score and duration-social and GRI in AOI-sociallost its significance exclusively in the lrADOS group (see OSFDR).

## 4. Discussion

The objective of the present study was to examine two types of social attention paradigms by analyzing specific eye tracking variables in toddlers below and above ADOS cut-off points. The main research question was whether eye movement patterns in these prevalent social attention paradigms could effectively differentiate toddlers with hrADOS from those in the elevated-likelihood sample. In order to achieve this objective, we employ both classic eye tracking variables (LRVT AOI) and our specially developed gaze retention interval (GRI) variables. Finally, the aim was to establish which variables demonstrate a strong correlation with autistic signs by means of ADOS.

First, we examined the differences between the hrADOS and lrADOS groups. As expected, we found significant differences between the two groups for most of the variables, although with different effect sizes. Typically, in the preferential paradigm, the distinct indicators of the social area were higher on average in the lrADOS group than in the hrADOS group, while the non-social area exhibited the opposite trend. These results are in accordance with the direction of previous studies [12,13,14,15,17], as well as with our hypotheses. In the joint attention paradigm, the group differences seemed to manifest in relation to the prompting and ostensive aspects of the person [7]. The hrADOS group consistently demonstrated a lower level of social attention towards the person, albeit with a smaller effect size compared to the preferential paradigm. The effect size results of the AOI ratio analysis indicate that the preferential paradigm is more promising in differentiating between the hrADOS and lrADOS groups; therefore, the new GRI variables were tested only in these data. The present findings suggest that the preferential paradigm contributes more to the differentiation of groups than joint attention stimuli. This finding suggests the presence of not only social attention but also more general differences in attention control processes [19], which are potentially better captured by the preferential paradigm. This claim is further supported by the findings of this study in non-social areas.

We examined the differences in the gaze retention interval (GRI) variables in the preferential paradigm, anticipating that certain variables would yield a larger effect size difference compared to the AOI ratio. According to the group comparisons of the GRI variables, the effect sizes of variables related to non-social area ranged between 0.66 and 1.19, while the effect size of variables related to social area ranged between 0.5 and 1.10. Similar to the variables based on AOI, the average for the hrADOS group was higher in the non-social variables compared to social variables. Therefore, no higher effect sizes are apparent in the differences observed between GRI variables. However, the values obtained with the density, GRI in AOI, and total gaze retention time variables confirm the difference between the groups obtained with AOI, especially with regard to the non-social area.

Our overarching objective was to identify eye tracking variables that potentially indicate the likelihood of ASD; therefore, it was fundamental for us to examine the relationship between our variables and the gold standard method for ASD diagnosis, the ADOS scores. We operate under the assumption that a linear relationship between the measured variables and the ADOS scores would validate that the observed group differences are indeed linked to autistic traits.

According to our results, regarding our AOI-based variables, the social ratio in the preferential paradigm had the strongest negative correlation with the ADOS score: the higher the toddler scored on ADOS, the less they spent looking at the social AOI area. We obtained a similar correlation with direct proportionality for the non-social area, and in both cases, this relationship proved to be significant with a higher effect size within the hrADOS group compared to the lrADOS group. Hence, our assumption that ADOS scores show a linear relationship with our variables is mostly valid within the hrADOS group. This coincides with the fact that the ADOS was created specifically for diagnostic purposes; thus, it is primarily for categorical separation, and, as it was indicated by earlier researches [33,34], it separates different levels of intensity within the group of diagnosed individuals.

With regard to GRI variables, those associated with the non-social area exhibit a positive correlation, whereas variables related to the social area display a negative correlation with the ADOS score. This was true of variables for which we had previously identified group differences. In addition, the correlation was more significant in the hrADOS group with the GRI variables, although, in several cases for the social area, it showed a significant correlation even within the lrADOS group (GRI in AOI—social; total gaze retention time—social; gaze stuck; and duration—social). In the hrADOS group, the most pronounced effect sizes were observed with variables related to the social domain; several GRI variables displayed a higher correlation coefficient compared to the AOI social ratio indicator (density—social; GR in AOI—social; GRI in AOI—non-social; duration—non-social; and total gaze retention time—non-social). The most noteworthy differences in effect size were identified within the HR ADOS group in the social area. Our results with GRI in AOI and density variables suggests that GRI provides a more refined measure of eye movement characteristics associated with autistic traits compared to classic AOI metrics. It is the opinion of the present authors that the findings support earlier suggestions that differences in eye movement during social attention cannot be explained solely by oculomotor differences [21].

### Limitation and Direction for Future Research

A limitation of the present study is that data concerning the clinical diagnosis of the participants at the time of data processing were not available. In Hungary, significant obstacles are present which hinder the timely diagnosis of patients. These include waiting lists which extend over several months due to a shortage of medical specialists and a lack of standardized diagnostic tools. However, individuals identified as potentially exhibiting autistic tendencies during ADOS evaluation were referred to a psychiatrist for further assessment. Participants will be followed up as our research continues. Consequently, the present findings can be considered as preliminary in regard to diagnostic predictions.

In the present analyses, the ADOS total score was applied; however, this reflects severity less accurately than the severity score proposed by Gotham et al. [33]. Esler et al. [35] specifically standardized the severity scores for the Toddler module. They emphasized that the implementation of severity scores reduces the impact of other characteristics of the children, thereby enabling more accurate conclusions to be drawn using such a variable.

A further limitation of our conclusions is that females are underrepresented in the sample, both in the total sample and in the hrADOS group. This phenomenon can be attributed, at least in part, to the prevalent assumption within society that autism is more commonly observed among the male population. Consequently, parents are more inclined to consider autism in boys than in girls for various behavioral indicators, in case of girls attributing different explanations to them [36]. However, a more pronounced discrepancy in the gender distribution is evident within the hrADOS group, a phenomenon that can be attributed to the fact that the ADOS method was predominantly designed for and tested on male subjects [37]. It is acknowledged that the current data do not permit conclusions about sex differences in eye tracking markers. This issue is also, at least in part, related to the recruitment process.

The decision to examine the elevated-likelihood sample was a deliberate choice and was not based on convenience. It is hypothesized that the discrepancies observed between toddlers who have already been diagnosed with autism and those who are developing typically do not necessarily facilitate the confirmation or refutation of suspected autism at a later stage using an eye-tracking screening method. A direct consequence of the sampling method employed is that a clear comparison between the groups and a typically developing group was not possible in our study. The primary objective of the recruitment process was to meet future goals.

Furthermore, the present study utilized a custom-designed eye tracker that was developed for the purpose of studying young children. Our device was specifically designed to accommodate young children by allowing for head movements and eliminating the need for calibration. While these characteristics may be suitable for toddlers, they may also introduce a potential bias in the estimation of gaze allocation, thereby influencing our outcomes. Regardless of our promising results, they should be further tested on more toddlers to replicate them.

## 5. Conclusions

In summary, we conducted testing on our innovative methodologies, e.g., stimuli and eye tracking variables, aimed at differentiating toddlers with hrADOS from an elevated-likelihood sample. In our study, we looked at two types of social attention paradigms and found that the preferential paradigm is more suitable for distinguishing toddlers with high risk of autism. Moreover, we employed the gaze retention interval (GRI), which encompasses a broader spatial and temporal range, rather than fixation. GRI variables proved to be good distinguishable variables.

We concluded that, in the preferential paradigm, apart from the AOI ratio, GRI variables can also prove effective in differentiating hrADOS toddlers. Additionally, they may be useful candidates for future predictive models of autism intensity and probability based on correlations with the ADOS. It is plausible that, when employed in combination, these variables may capture characteristics indicative of higher ADOS scores in toddlers. We anticipate that these results will contribute towards the development of screening tools for the early identification of autism. The necessity for prospective validation and diagnostic follow-up is crucial in this context.

## Figures and Tables

**Table 1:** Descriptive statistics of the study population.

		All					Males					Females				
		N	Mean	SD	Min	Max	N	Mean	SD	Min	Max	N	Mean	SD	Min	Max
Age (months)	ALL	74	22.8	4.9	12	30	49	23.1	5.1	12	30	25	22.2	4.8	15	30
	lrADOS	42	22.4	5.0	12	29	25	23.2	4.9	12	29	17	21.3	5.2	15	29
	hrADOS	32	23.3	4.8	12	30	24	23.0	5.3	12	30	8	24.3	3.2	19	30
ADOS score	ALL	74	9.5	7.6	0	25	49	10.8	7.8	0	25	25	6.8	6.6	0	24
	lrADOS	42	3.6	2.4	0	8	25	4.1	2.2	0	8	17	3.0	2.5	0	7
	hrADOS	32	17.1	4.9	10	25	24	17.9	4.7	10	25	8	14.8	5.3	10	24

**Table 2:** Test results of independent samples and effect sizes of eye movement variables in preferential and ostensive situations.

	Mean hrADOS	Mean lrADOS	*p*	Effect Size (Hedges’s g)
Preferential—ratio of non-social	***28.8%***	***17.3%***	0.0000 *	***1.11***
Preferential—ratio of social	***51.9%***	***67.4%***	0.0001 *	***1.00***
Ostensive—ratio of person (W)	**46.9%**	**58.3%**	**0.0429**	**0.50**
Ostensive—ratio of target (MW)	13.3%	13.2%	0.6931	0.05

Values in bold represent significant differences, and values in italics indicate large Hedge’s g effect sizes. * These significant results survived the multiple-comparison correction. (W—Welch test; MW—Mann–Whitney test).

**Table 3:** Test results of independent samples and effect sizes of GRI (gaze retention interval) variables in preferential situations.

		Mean hrADOS	Mean lrADOS	*p*	Effect Size (Hedge’s g)
Density (1/min)	Non-social (MW)	***20.9***	***12.7***	0.0000 *	***0.55***
	Social (W)	***32.5***	***38.1***	0.0108 *	***0.63***
Duration (ms)	Non-social (MW)	763.4	747.0	0.9180	0.15
	Social	999.4	1076.2	0.3203	0.23
Gaze retention in AOI (%)	Non-social (W)	***34.2%***	***23.0%***	0.0001 *	***1.01***
	Social (W)	***56.6%***	***70.5%***	0.0000 *	***1.04***
Total gaze retention time (ms)	Non-social (MW)	***2361.3***	***1363.5***	0.0000 *	***0.56***
	Social	***4308.6***	***5534.3***	0.0004 *	***0.87***
Latency (ms)	Non-social	1644.2	1616.3	0.8596	0.04
	Social	1151.1	1020.9	0.2000	0.30
Gaze stuck (ms)	Non-social (MW)	***123.6***	***62.1***	0.0074 *	***0.36***
	Social	**232.9**	**311.8**	**0.0239**	**0.54**

Values in bold represent significant differences, and values in italics indicate large Hedge’s g effect sizes. (W—Welch test; MW—Mann–Whitney test). For the 95% confidence interval of the group means, see the OSFDR. * These significant results survived the multiple-comparison correction.

**Table 4:** Correlations between ADOS scores and AOI variables in preferential and ostensive situations.

	ALL		lrADOS		hrADOS		Comparison of Correlations	
	r *	*p*	r	*p*	r	*p*	Fisher’s z	*p*
Preferential—ratio of non-social	**0.59**	**0.0000**	0.09	0.5677	**0.51**	**0.0030**	−1.90	0.0574
Preferential—ratio of social	**−0.63**	**0.0000**	**−0.35**	**0.0250**	**−0.66**	**0.0000**	1.74	0.0818
Ostensive—ratio of person	**−0.41**	**0.0004**	−0.30	0.0569	**−0.45**	**0.0092**	0.73	0.4654
Ostensive—ratio of target (SP)	0.01	0.9388	0.17	0.2936	0.04	0.8390	0.53	0.2970

* In each column, the r and *p* values of the correlations between ADOS score and eye movement variables are presented for all subjects (hrADOS and lrADOS together), for lrADOS subjects and for hrADOS subjects. The last two columns show the difference (expressed in Fisher’s z score) between the correlations of lrADOS and hrADOS subjects and the significance of the difference. Values in bold represent significant correlations/differences. (SP—Spearman correlation).

**Table 5:** Correlations between ADOS scores and gaze retention interval (GRI) variables in preferential situations.

		ALL		lrADOS		hrADOS		Comparison of Correlations	
		r *	*p*	r	*p*	r	*p*	Fisher’s z	*p*
Density (1/min)	Non-social	**0.58**	**0.0000**	0.11	0.4830	**0.41**	**0.0185**	−1.34	0.180
	Social	**−0.52**	**0.0000**	0.00	0.9935	**−0.77**	**0.0000**	**4.12**	**0.000**
Duration (ms)	Non-social (SP)	0.07	0.5426	−0.17	0.2704	**0.60**	**0.0003**	**−3.08**	**0.002**
	Social	−0.21	0.0733	**−0.35**	**0.0244**	−0.17	0.3509	−0.77	0.438
Gaze retention in AOI (%)	Non-social	**0.60**	**0.0000**	0.14	0.3839	**0.57**	**0.0007**	**−2.07**	**0.038**
	Social	**−0.68**	**0.0000**	**−0.36**	**0.0181**	**−0.71**	**0.0000**	**2.03**	**0.042**
Total gaze retention time (ms)	Non-social	**0.60**	**0.0000**	0.05	0.7433	**0.54**	**0.0014**	**−2.26**	**0.024**
	Social	**−0.58**	**0.0000**	**−0.38**	**0.0125**	**−0.63**	**0.0001**	1.37	0.172
Latency (ms)	Non-social	−0.13	0.2801	−0.09	0.5831	**−0.44**	**0.0122**	1.56	0.120
	Social	**0.30**	**0.0102**	0.07	0.6467	**0.49**	**0.0041**	−1.91	0.056
Gaze stuck (ms)	Non-social (SP)	**0.37**	**0.0001**	0.11	0.4675	**0.38**	**0.0297**	−1.71	0.086
	Social	**−0.41**	**0.0003**	**−0.38**	**0.0126**	**−0.42**	**0.0166**	0.19	0.852

* In each column, the r and *p* values of the correlations between ADOS score and eye movement variables are presented for all subjects (hrADOS and lrADOS together), for lrADOS subjects and for hrADOS subjects. The last two columns show the difference (expressed in Fisher’s z score) between the correlations of lrADOS and hrADOS subjects and the significance of the difference. Values in bold represent significant correlations/differences. (SP—Spearman correlation).

## Data Availability

The datasets generated and/or analyzed during the current study are available in the OSF data repository [https://osf.io/hvxu9/?view_only=30732c28ec094e1eba87a9ede464a002].

## Abbreviations

The following abbreviations are used in this manuscript:

ADOS	Autism Diagnostic Observation Schedule
AOI	Area of Interest
ASD	Autism Spectrum Disorder
GRI	Gaze Retention Interval
IDT	Identification by Dispersion Threshold
OSFDR	OSF Data Repository
